# Chemically activated core–shell structured IF-WS_2_@C nanoparticles enhance sugarcane-based carbon/epoxy nanocomposites

**DOI:** 10.1039/d1ra07136j

**Published:** 2021-11-17

**Authors:** Dehua Cao, Guangsheng Liu, Wenting Chen, Xuefeng Lv, Taize Song, Linyi Zhang, Song Liu, Yi Li, Nannan Wang, Yanqiu Zhu

**Affiliations:** Key Laboratory of New Processing Technology for Nonferrous Metals and Materials, Ministry of Education, Guangxi Institute Fullerene Technology (GIFT), School of Resources, Environment and Materials, Guangxi University Nanning 530004 China wangnannan@gxu.edu.cn; College of Engineering, Mathematics and Physical Sciences, University of Exeter EX4 4QF UK

## Abstract

Ternary composites have demonstrated better capability than binary composites in enhancing the mechanical properties of the modified epoxy resins and are, therefore, currently under intensive investigation. Herein, we report a novel ternary nanocomposite prepared by filling a binary BPF (bisphenol F epoxy resin)/SCPs (sugarcane-based carbon powders) matrix with C-coated inorganic fullerene-like tungsten disulfide (IF-WS_2_@C) nanoparticles, and the analysis of its interface synergetic effect using XPS/FTIR. This activated nano-carbon core–shell structure filler is considered an ideal nanofiller and shows the excellent mechanical performance of the ternary composites. XRD, IR, XPS, SEM, and TEM characterizations were applied in investigating this nanocomposite. The improvement of the thermal and mechanical properties demonstrated the enhancement effects of this nanofiller. The results show that the great improvement of the bending modulus of 39.4% increased with the addition of 0.5 wt% IF-WS_2_@C nanoparticles, while 34.1% enhancement of bending strength was obtained with the addition of 0.2 wt% IF-WS_2_@C nanoparticles. The hardness and thermal conductivity were also boosted up to 5.2% and 33.1% with 0.5 wt% addition, respectively. The incorporation of a chemically activated coating may provide a novel means for improving graphite crystallization, which could somehow expand the potential application of IF-WS_2_@C nanoparticles.

## Abstract 2

Schematic diagram and typical curing mechanism of epoxy resins and the unique interactions of the IF-WS_2_@C nanoparticles introduced into the matrix.

## Graphical abstract

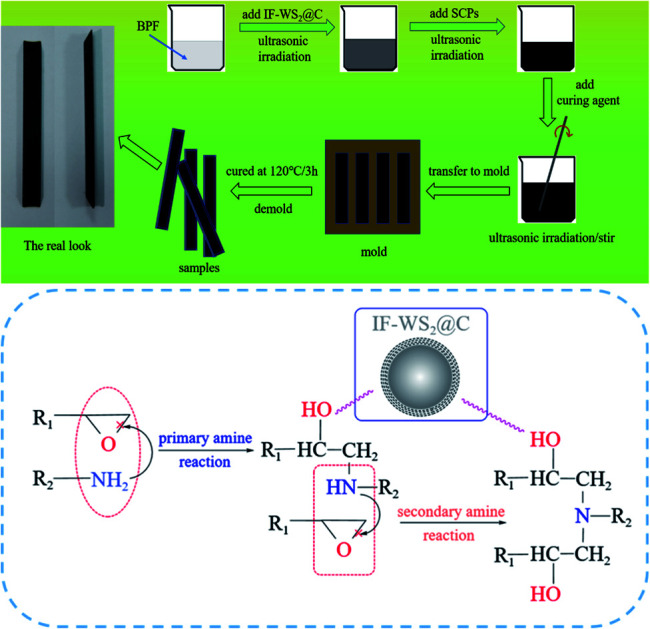

## 1. Introduction

For decades, hybrid inorganic–organic polymer-based materials have been reported for their excellent performances that are ascribed to the combination of the advantages of both the organic polymer matrix and inorganic materials.^1^ The physical properties of polymers, such as dielectric,^2,3^ mechanical,^4^ and thermal conductivity,^5^ have been widely researched and new features have been implemented in the polymer matrix by adding inorganic nanoparticles to the polymers. These composite materials affect several fields of application, namely aerospace structure, biological medicine, and other industrial fields.^6^

To date, many studies have been reported on core–shell structure composites. Zhou *et al.*^7^ synthesized and investigated core–shell structured SiO_2_-coated polystyrene (PS@SiO_2_) microspheres and the results showed that the thermal conductivity increased by 131%, as well as the dielectric constant, with the addition of 1 wt% PS@SiO_2_ microspheres into the epoxy resin. Chen *et al.*^8^ reported a novel manufacturing process, laser sintering, to produce nanocomposite powders and fabricate core–shell structured polyetherimide (PEI)-coated polyether–ether–ketone (PEEK) and WS_2_-PEI coated PEEK composites. Their study revealed that the powder rheology properties and mechanical performances were considerably enhanced. The core–shell structured calcium silicate composite filler studied by Li *et al.*^9^ demonstrated its capability in increasing the tensile and tear indices of hand sheets. The superiorities of core–shell structured particles for improving the composite performances were clearly evidenced.

Recently, due to the discovery of IF-WS_2_, Tenne *et al.*^10^ has opened up a whole new field of research and garnered considerable academic interest from many researchers. The so-called IF-WS_2_ was formed by bending layers into a closed-cage carbon fullerene-like structure under certain conditions and hence favorable stability could be obtained. IF-WS_2_ also exhibits superb tribological properties and shock absorbing behavior due to its unique chemical bonding structure, which contributes significantly to the improvement of tribological and mechanical properties in composites.^11–14^ For example, improved properties, with the addition of IF-WS_2_ nanoparticles, have been frequently observed in polymers such as epoxy,^15,16^ PEEK,^17–19^ nylon-6,^20^ PPS,^21^*etc.* Molecular modifications of the IF-WS_2_ surface have also been tried in many studies. Shahar *et al.*^22^ reported that the modified IF-WS_2_, coated with alkyl-silane derivatives with Si atoms attached to the surface, improved the oil's long-term tribological behavior and reduced the agglomerating tendency of IF-WS_2_ nanoparticles. Xu *et al.*^23^ synthesized carbon-coated IF-WS_2_ nanoparticles with improved thermal properties, which is significant progress that inspired us to explore performance enhancement in composites.

Epoxy resin (ER) is a kind of thermosetting resin with various forms and easy curing capability. The cured ER has the characteristics of strong adhesion, low shrinkage, excellent chemical and electrical properties, and strong chemical stability, which allow it to be widely used in the machinery, motor, chemical industry, aerospace, automotive, construction, and other industrial sectors.^3,24–27^ Researchers found that incorporating a second phase component into ER might overcome the drawbacks of its inherent brittleness that restricts its wide applications. SCP, a renewable biomass material that features non-toxicity, eco-friendliness, and ultra-lightness, has good compatibility with most matrices as a filler in composites. Monteiro *et al.*^28^ studied the incorporation of bagasse into ER for ballistic-resistant materials and demonstrated that the composites could replace Kevlar for multilayered armor application for the first time. Pan *et al.*^29^ prepared a wood–ceramic using sugarcane bagasse and ER that has the potential for application as catalyst supports, filters, and absorbents.

Applications of nanocarbon/ER binary composites are also of growing interest in materials industries for improving the material properties to meet market demands.^30,31^ For example, Yang *et al.*^32^ discovered that the thermal conductivity of the ER had dramatically improved (about 684% of the original) after filling the matrix with functionalized carbon nanotubes. Liu *et al.*^33^ also investigated a composite material with high toughness and strain sensitivity. The composite was prepared by filling symmetric plasma-functionalized carbon nanotube films into ER, and the obtained composite material exhibited better peeling strength and tensile properties.

Overall, carbon/ER composites and core–shell structured nanoparticles/ER composites with various improved performances have been widely studied so far. However, using core–shell structured materials, *i.e.*, C-coated IF-WS_2_ nanoparticles, as fillers for the performance enhancement of nanocarbon/epoxy composites has not been reported yet. The synergetic effect of IF-WS_2_@C and SCPs may drive the property enhancement in ternary composite, according to some reports.^34,35^ This study reveals the possibility of applying core–shell structured IF-WS_2_ nanoparticles to the nanocarbon/epoxy composites to obtain performance-enhanced materials.

## 2. Experimental

### 2.1 Materials

All raw materials used in this study are divided into three categories: (1) matrix; (2) fillers; (3) curing agent. The polymer matrix, bisphenol F epoxy resin, and the amide curing agent were supplied by Changzhou Advanced Materials Research Institute, Beijing University of Chemical Technology (purchased from Dalian Liansheng Trading Co. Ltd). C-coated IF-WS_2_ nanoparticles were prepared by a chemical vapor deposition (CVD) method^23^ and supplied by the College of Engineering, Mathematics and Physical Sciences, University of Exeter, UK. In a typical process, nanosized WO_3_ powders were put into a rotary furnace and reacted with H_2_S under an argon atmosphere at 800 °C to produce IF-WS_2_. Afterwards, the H_2_S gas switch was closed and the carbon sources (mixture of styrene and acetone) were injected at a constant rate. After reacting for several hours, the obtained products were the C-coated IF-WS_2_ nanoparticles. Sugarcane bagasse was obtained locally as a carbon powder source; both serve as fillers for the composite materials.

### 2.2 Fabrication of IF-WS_2_@C/SCPs/BPF composites

**Fig. 1:**
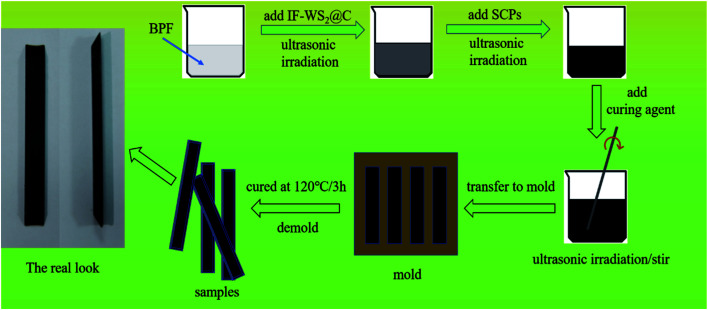
Schematic diagram of the process for the synthesis of ternary composites.

The IF-WS_2_@C/SCPs/BPF composites were prepared by dispersing 0.02, 0.04, or 0.1 g of IF-WS_2_@C nanoparticles in 19.8 g bisphenol-F epoxy resin in an ultrasonic oscillator for 3 h, followed by adding 0.2 g of SCPs with an extra 1 h of ultrasonic dispersion. Then, 5.94 g of curing agent was added and the mixture was stirred mechanically for 30 minutes. After ultrasonic irradiation one more time (to remove bubbles as much as possible), the prepared composite precursor was poured into a trapezoidal spline mold of 90 mm in length, 10 mm in width, and 4 mm in thickness, and aged at room temperature for 10 min. Finally, the samples were dried in an oven at 120 °C for 3 h and cooled to room temperature for performance evaluation. A binary composite of SCPs/BPF with no IF-WS_2_@C nanoparticles was prepared in the same way for comparison. A schematic diagram of the process for the synthesis of ternary composites is presented in Fig. 1.

### 2.3 Characterizations and measurements

The powder X-ray diffraction (XRD) patterns of the composites were recorded using an X-ray diffractometer (Rigaku D/MAX 2500 V) with Cu-Kα radiation (*λ* = 0.154056 nm) at 40 kV and 30 mA in the range of 10–80°. The continuous scanning mode was set at a step size of 0.02° and a step time of 1 s. Characterization of the isolated C-coated IF-WS_2_ nanoparticles was performed after evenly spreading the materials on a concave slide.

Transmission electron microscopy (TEM) images of IF-WS_2_@C nanoparticles were obtained using a JEM-2100 instrument operating at an accelerating voltage of 200 kV. An Extreme-resolution Analytical Field Emission Scanning Electron Microscope (SEM, Tescan Mira 3 XH) was used to observe the cross-sectional morphology of IF-WS_2_@C/SCPs/BPF composites at an operational voltage of 5 kV. The elemental distribution was further analyzed by EDS (Aztec X-MaxN 80). All the composite specimens were manually fractured after liquid nitrogen cooling-break processing and spray-coated with gold prior to the SEM inspection.

The structural characteristics and functionalities of bisphenol-F epoxy resin and the ternary composites used in this study were analyzed by attenuated total reflection Fourier transform infrared spectroscopy (ATR-FTIR) in the scanning range of 400–4000 cm^−1^.

X-ray photoelectron spectroscopy (XPS, Thermo Fisher, ESCALAB 250Xi) was adopted for exploring the mechanism of the interface bonding between the matrix and the fillers, using Al Kα X-ray (*hν* = 1486.6 eV) as the excitation source with 12.5 kV charging voltage and 16 mA emission current.

Instrumented indentation testing (IIT) with the KLA Nano Indenter® system was employed to determine the elastic modulus and hardness of binary and ternary composites, allowing the extraction of abundant information from the force-displacement records during the experiment. Here, 64 points were measured for each sample by setting up an 8 × 8 array in the testing system; mechanical properties were characterized by these two values in IIT.

The thermal conductivity of the ternary composites was measured by a NETZSCH LFA 467 Hype Flash between −25 and 100 °C at an interval of 25 °C. The corresponding thermal conductivity was obtained by averaging the results of 3 repeated measurements at each temperature.

## 3. Results and discussion

### 3.1 Morphology and structural characterization

**Fig. 2:**
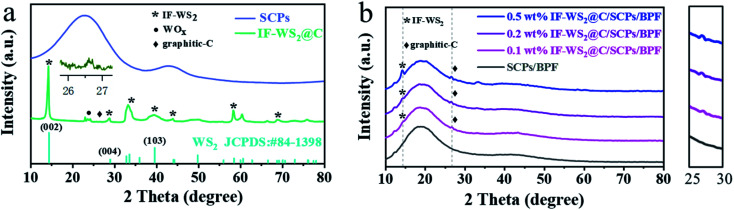
XRD patterns of (a) pure sugarcane-based carbon particles (SCPs) and IF-WS_2_@C nanoparticles, (b) SCPs/BPF composites with different contents of IF-WS_2_@C nanoparticles (0, 0.1, 0.2, and 0.5 wt%).

The XRD patterns of IF-WS_2_@C nanoparticles, SCPs, and the binary composites with different IF-WS_2_@C contents are shown in Fig. 2(a) and (b). As presented in Fig. 2(a), IF-WS_2_@C nanoparticles show a significant crystallographic diffraction pattern with an intense peak at 14.3° belonging to IF-WS_2_ (ref. 23), which corresponds to the (002) plane of WS_2_ (JCPDS no. 84-1398). The SCPs/BPF composite matrix displayed amorphous behavior with broad peaks around 19° and 42°, as shown in Fig. 2(b). With the increment of the IF-WS_2_@C nanoparticles in the SCPs/BPF composites, the corresponding diffraction intensity of the fillers became more visible. This indicates that IF-WS_2_@C nanoparticles were introduced into the composites. A close look at the diffraction patterns of IF-WS_2_@C-coated composites revealed weak diffraction reflections around 26.6°, which belong to graphite carbon. Meanwhile, the corresponding intensities seemed to increase and were still located in the same position with further addition of IF-WS_2_@C in the ternary composites. Comparatively, a very weak graphite XRD reflection was observed in the IF-WS_2_@C sample. It is therefore postulated that the graphitic carbon coating on the nanoparticles may serve as crystal seeds to trigger the partial crystallization of the amorphous form of carbon in the composites during the cooling process after a high-temperature curing process. If this is the case, this phenomenon may suggest a novel pathway for improving the crystallization degree of amorphous carbon.

**Fig. 3:**
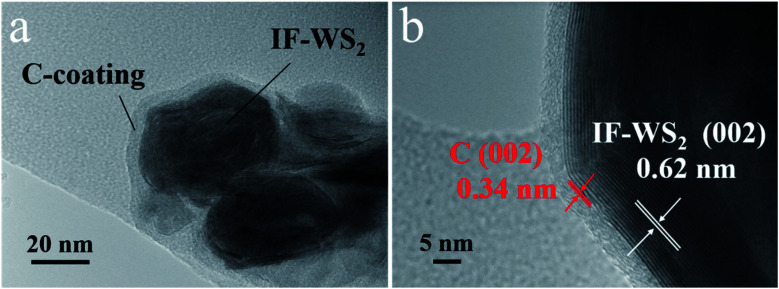
(a) TEM and (b) HRTEM images of IF-WS_2_@C nanoparticles.

Fig. 3(a) and (b) present the detailed structure and crystallographic features of granular IF-WS_2_@C nanoparticles in TEM images. Fig. 3(a) shows the core–shell-structured IF-WS_2_@C nanoparticle in possession of a dark core and a shallow shell that could be ascribed to IF-WS_2_ and graphitic carbon, respectively. The high-resolution TEM (HRTEM, Fig. 3(b)) of the core–shell interface further confirmed a typical 0.34 nm distance for the (002) crystallographic planes, which comes from the graphitic carbon coating. The IF-WS_2_ core shows the 0.62 nm assigned to the (002) planes. The core IF-WS_2_ nanoparticles with non-uniform shapes were roughly 20–50 nm in diameter while the carbon coatings had a uniform thickness of 4–5 nm, which originated from the pyrolysis of hydrocarbons or other carbon sources during CVD fabrication. These thin graphite coatings correspond to the weak peak at 26.6° obtained in the XRD pattern; the graphite coating enabled the thermal stability and thermal conductivity.^36^

**Fig. 4:**
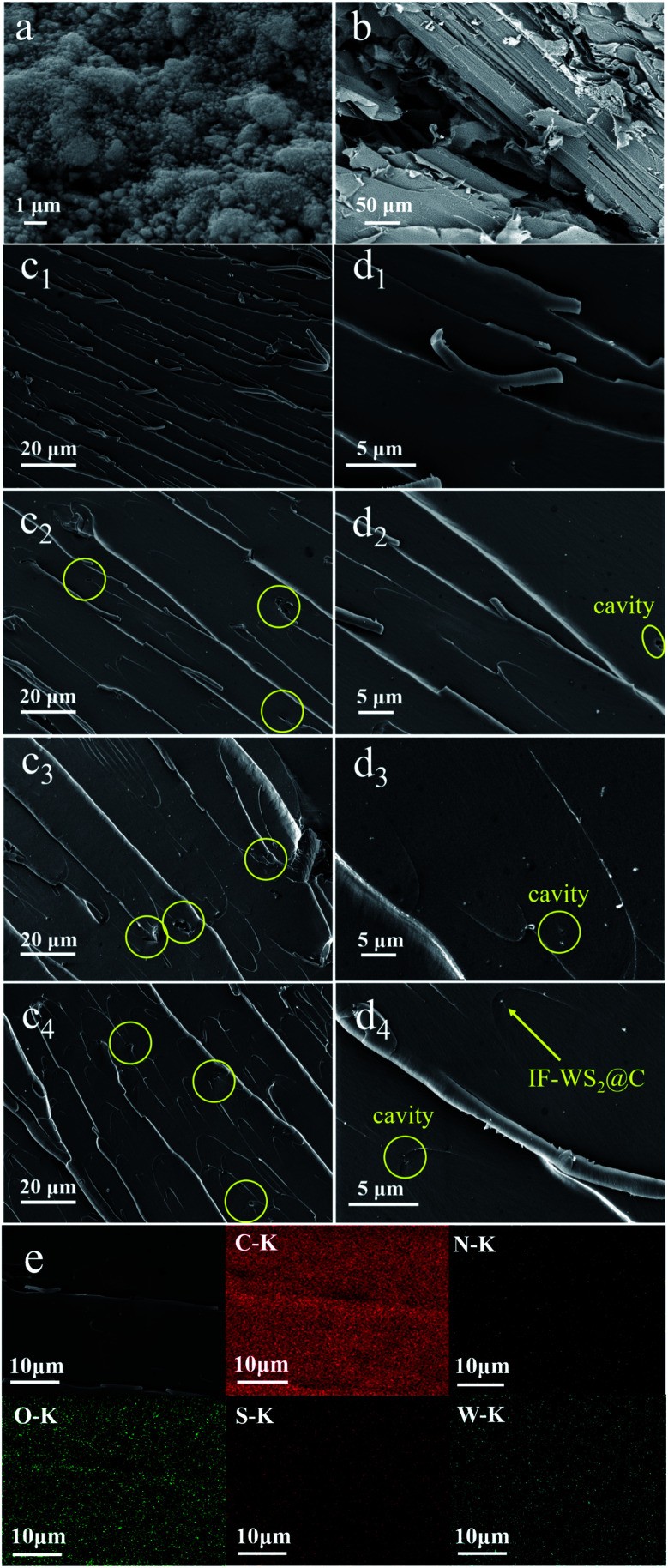
SEM images of (a) IF-WS_2_@C nanoparticles and (b) sugarcane-based carbon powders (SCPs). (c_1_–c_4_ and d_1_–d_4_) The fracture morphologies of IF-WS_2_@C/SCPs/BPF composites with different contents of IF-WS_2_@C nanoparticles (0 wt%, 0.1 wt%, 0.2 wt%, 0.5 wt%) at different magnifications. (e) The elemental mappings of cross-sectional SEM images of ternary composites.

SEM images of IF-WS_2_@C nanoparticles, SCPs, and the fracture morphology of composites are displayed in Fig. 4. As seen in Fig. 4(a), the IF-WS_2_@C nanoparticles tend to agglomerate due to the nano-size effect and show a bright appearance. A typical wood fiber of SCPs with some intrinsic features is presented in Fig. 4(b). The SEM images in Fig. 4(c) and (d) are the fracture morphologies of the ternary composites with diverse filling contents of IF-WS_2_@C nanoparticles under different magnifications. According to Fig. 4(c_1_) and (d_1_), some sliver lines, and relatively neat fracture can be observed in the SCPs/BPF binary composites, which suggest weak resistance to the generation and extension of the cracks.^34^ In contrast, increasingly twisted river-like lines and several cavities appeared on the fracture surface of IF-WS_2_@C/SCPs/BPF ternary composites. These morphologies have been reported as the signs of strengthening effect in composites.^16,37^ In detail, the twisted crack lines occur when the hard filler particles impede the crack propagation. The observed cavities (circled in yellow in Fig. 4(c_2_–c_4_) and (d_2_–d_4_)) were considered as the consequence of particles drifting. Moreover, IF-WS_2_@C nanoparticles, incorporated within binary composites, seemed to embody a pinning effect on crack lines similar to spherulites. Overall, it is believed that the introduced IF-WS_2_@C nanoparticles were well-dispersed within the SCPs/BPF matrix, which was further demonstrated by the elemental distribution mappings of cross-sectional SEM images of ternary composites presented in Fig. 4(e), and good matrix/filler interfaces were generated. Based on the variation in the cracks and matrix/filler interface in the ternary SCPs/BPF matrix with the IF-WS_2_@C content, it is reasonable to deduce that the introduced IF-WS_2_@C nanoparticles may benefit the property improvement in SCPs/BPF composites.

**Fig. 5:**
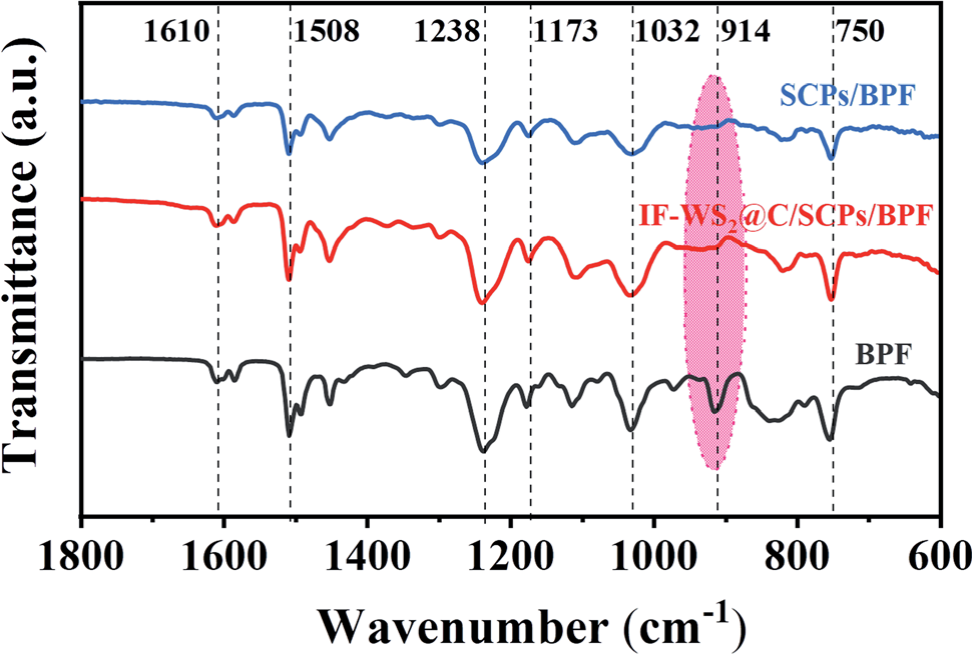
IR spectra of BPF, SCPs/BPF, and the IF-WS_2_@C/SCPs/BPF composite.

The IR spectra of the BPF filled with fillers are shown in Fig. 5. As depicted, most peaks fit well with the molecular structure of BPF. However, the typical peak around 914 cm^−1^ belonging to epoxide groups disappeared, which indicated their consumption during the reaction of BPF and curing agent. The absorption peak around 1610 and 1508 cm^−1^ was attributed to the typical vibration of the benzene ring skeleton in the epoxy matrix. The peak at around 1238 cm^−1^ originated from the stretching vibration of aromatic C–O, representing a direct linkage of the aromatic carbon with the oxygen atom of the aromatic polyether; the stretching vibration of the branched C–O peak appeared at 1032 cm^−1^ was also derived from the aromatic polyether. Absorption peaks at 1173 and 750 cm^−1^ were assigned to the stretching vibration and out-plane flexural vibration of the amino group (–NH_2_). The FTIR results suggest that the introduced IF-WS_2_@C nanoparticles did not damage the structure of BPF in the ternary composite. Nevertheless, the bonding evolutions and quantitative information in the composites followed by the addition of IF-WS_2_@C nanoparticles will be further explained by the XPS analysis.

**Fig. 6:**
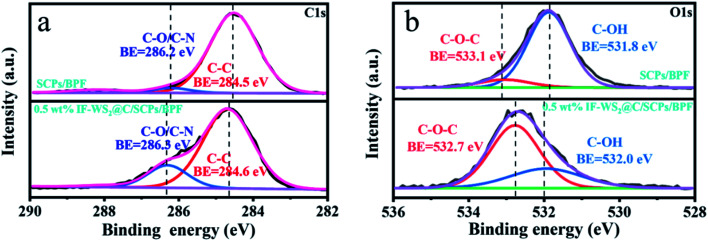
(a) C 1s XPS survey spectra of SCPs/BPF and the 0.5 wt% IF-WS_2_@C/SCPs/BPF composite; (b) O 1s XPS survey spectra of SCPs/BPF and the 0.5 wt% IF-WS_2_@C/SCPs/BPF composite.

XPS analysis was carried out and the XPS survey of the binary and ternary composites are presented in Fig. 6(a) and (b). The broad and asymmetric C 1s XPS spectrum was further deconvoluted into major peaks centered at 284.6 and 286.2 eV, corresponding to the C–C and C–O or C–N bonding, respectively, as presented in Fig. 6(a). Meanwhile, Fig. 6(b) shows that the O 1s spectra for two different samples were fitted to two peaks as follows: C–OH (532 eV) and C–O–C (533 eV). No significant difference was found in the intensity of C–C bonding between the composites when the peak area of C–O/C–N was much bigger according to the C 1s spectra, and the peak area of C–O–C became broader while the peak area of C–OH became smaller based on the O 1s spectra. The locations of the related binding energies also shifted a little. Considering the curing mechanism of ER, primary and secondary crosslinking reactions occurred between the epoxide groups from BPF and amino groups from the curing agent under the curing temperature, resulting in the formation of C–OH groups. Variations in the peak area based on C–N/C–O in the C 1s spectra and C–O–C/C–OH in the O 1s spectra may be reasonably explained by the interaction of the C–OH groups and carbon shell coated on IF-WS_2_ nanoparticles, *i.e.*, the transfer of the H-proton. This kind of protonic transfer involved in C–OH bonding promoted the increase in the amount of C–O bonding favored by the heat curing. Such a case further promoted the orderly arrangement of molecular chains, supported by the differences in the related peak areas and was corroborated by the improvement in the graphitic crystallinity suggested in XRD results. Therefore, it is reasonable to believe that the graphitic shell coated on IF-WS_2_ nanoparticles has chemically activated reactivity that is linked to the epoxy network. Such evolution implies that the IF-WS_2_@C nanoparticles were perfectly integrated into the BPF matrix, and therefore developed an effect to enhance the mechanical and thermal properties of the ternary composites.

**Fig. 7:**
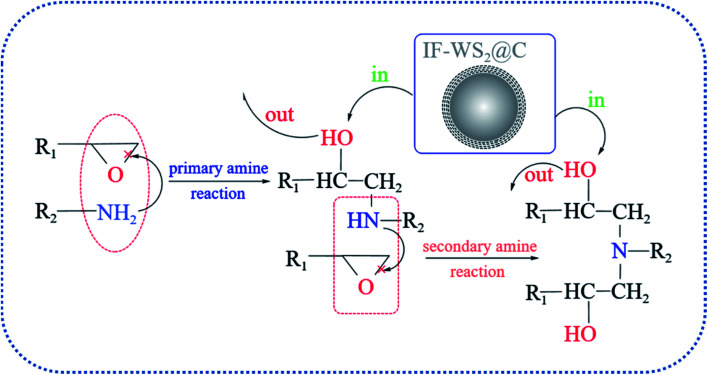
Typical curing mechanism of epoxy resins and the unique interaction of the introduced IF-WS_2_@C nanoparticles with the matrix.

The structural characterizations mentioned above corroborate the behavior of introducing IF-WS_2_@C nanoparticles into the BPF matrix beyond intrinsic expectation. An illustration of the reaction mechanism is plotted in Fig. 7 according to a series of structural evolutions, which revealed the unique interaction of the incorporated IF-WS_2_@C nanoparticles and the matrix after primary and secondary amine reactions. The results show that not only is the original structure of the matrix network not destroyed but it also can be combined with the BPF matrix, even triggering partial crystallization of the amorphous form of carbon in the composites. As a result, it can be predicted rationally that the introduction of IF-WS_2_@C nanoparticles will affect the thermal and mechanical properties of SCPs/BPF composites.

### 3.2 Mechanical properties

**Fig. 8:**
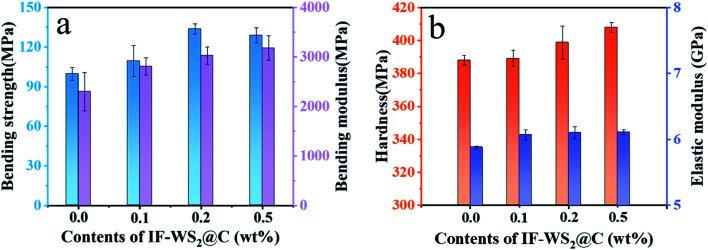
(a) Bending strength and modulus results. (b) Hardness and elastic modulus results of IF-WS_2_@C/SCPs/BPF ternary composites with different IF-WS_2_@C contents (0, 0.1, 0.2, and 0.5 wt%).

Fig. 8(a) illustrates the relationship between the bending strength and modulus of composites and the content of added nanofiller in the composites. Based on the bending strength, 99.7 MPa, of the pristine SCPs/BPF binary composite, the optimal bending strength, 133.7 MPa, was obtained when introducing 0.2 wt% IF-WS_2_@C nanoparticles into the SCPs/BPF binary composite, and thus 34.1% improvement was achieved. Mixing excessive heterogeneous nanoparticles in the polymer matrix may cause defect formation and stress concentration, whereas adding a small number of nanoparticles into the matrix may benefit the mechanical properties. Slightly differing in the strength trends, it was found that the bending modulus increased by 23.2%, 32.9% and 39.4%, respectively, when 0.1 wt%, 0.2 wt% and 0.5 wt% of IF-WS_2_@C nanoparticles were added as compared to the pristine SCPs/BPF binary composite. Therefore, the introduction of rigid IF-WS_2_@C nanoparticles into the BPF system did not cause damage to the internal structure but rather enhanced the atomic bonding and reduced the presence of bubbles in the BPF. For a direct comparison, the bending properties of related sugarcane-bagasse-filled ER composites are listed in Table 1.

**Table 1:** Comparison of the bending properties of various sugarcane–epoxy composites

Composites	Bending strength (MPa)	Bending modulus (MPa)
30 vol% SBF/ER^38^	<30	—
30 wt% SBF/ER^39^	45.34	—
6 vol% SDL/ER^40^	51.47	—
30 wt% SBF/BPF^41^	73.5	2301.3
This work	133.7	3029.8

Instrumented indentation testing (IIT) with the KLA Nano Indenter® systems was employed to investigate the hardness and elastic modulus of the as-prepared composites. Fig. 8(b) shows the hardness of the composites filled with different amounts of IF-WS_2_@C nanoparticles from 0 to 0.5 wt%. The results indicate that the hardness of the specimen increased with the addition of IF-WS_2_@C to the composite materials. The greatest improvement of hardness was obtained when 0.5 wt% IF-WS_2_@C nanoparticles were added to the SCPs/BPF binary composites, from 388 MPa to 408 MPa, which achieved a 5.2% increase as compared to the pure binary composite. This confirmed that the rigid IF-WS_2_@C nanoparticles stiffened the matrix, which can be ascribed to their unique onion-like structure and strain mechanism. The direct stress may weaken layer by layer through IF-WS_2_@C nanoparticles, and the carbon coating may play the role of a buffer layer. The elastic modulus continued to increase with increasing the content of IF-WS_2_@C nanoparticles; the maximum modulus of 6.11 GPa was obtained when 0.5 wt% IF-WS_2_@C nanoparticles were added to SCPs/BPF binary composites, based on the 5.88 GPa modulus of the pristine binary composite. As characterized above, it is believed that the well-dispersed IF-WS_2_@C particles were closely bound with the matrix both chemically and physically, and hence the enhancement of physical properties was achieved.

**Fig. 9:**
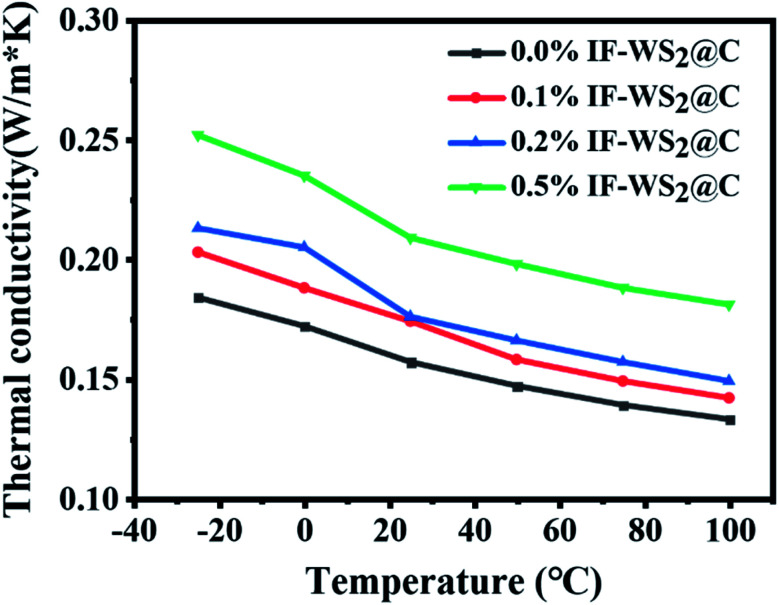
Thermal conductivity of ternary IF-WS_2_@C/SCPs/BPF with different IF-WS_2_@C contents (0, 0.1, 0.2, and 0.5 wt%).

The thermal conductivities of the composites with different IF-WS_2_ @C contents, evaluated from −25 to 100 °C, are shown in Fig. 9. The thermal conductivity of the binary SCPs/BPF composite at room temperature was roughly 0.157 W m^−1^ K^−1^, and 10.8%, 11.2%, and 33.1% improvements were obtained for the composites with 0.1 wt%, 0.2 wt%, and 0.5 wt% additions of IF-WS_2_@C nanoparticles. This demonstrates that a small amount of IF-WS_2_@C nanoparticles can enhance the thermal conductivity of the binary SCPs/BPF composites to some extent. The incorporation of well-dispersed IF-WS_2_@C nanoparticles with the BPF matrix is assumed to provide good pathways for heat conduction in the composites. On the other hand, with the increase in the testing temperature, the thermal conductivity of all composites displayed a downward trend. This could be explained by the combined action of nanoparticle desolation and debonding of the epoxy network, which destroyed the heat conduction pathways. The overall improvement of the thermal conductivity of the composites with the addition of IF-WS_2_@C nanoparticles seems to be more significant, even though IF-WS_2_@C nanoparticles possess ultra-high thermal conductivity. This might be related to the interfacial thermal resistance between the filler nanoparticles and the composite matrix. The incorporated nanoparticles can increase the phonon scattering as well, which can introduce additional adverse effects on heat conduction. Nevertheless, other factors, such as volume-weight, coefficient of linear expansion, the direction of thermal flux, and so on, should not be excluded from accounting for this limited enhancement effect of nanofiller in composites.

## 4. Conclusions

Epoxy-based ternary composites filled with IF-WS_2_@C and SCPs exhibited improved properties that combined the advantages of both the organic polymer matrix and inorganic materials. The better dispersion was formed between the C-coated IF-WS_2_ nanoparticles and sugarcane-based carbon powders integrated within the epoxy matrix. The combination of chemical bonding and physical filling resulted in improved mechanical and thermal properties. Enhancements of 39.4% in the bending modulus, 5.2% in the hardness, and 33.1% in the thermal conductivity were achieved by the incorporation of 0.5 wt% IF-WS_2_@C nanoparticles into the composites, while 34.1% improvement of bending strength was obtained with the addition of 0.2 wt% IF-WS_2_@C nanoparticles. This demonstrates that introducing second special nanofillers in composites can practically lead to better composite properties than simple binary composites since the core–shell structured IF-WS_2_@C nanoparticles with chemically activated coating exhibited excellent performance within the epoxy. Besides, the incorporation of a chemically activated coating may provide a novel means for improving graphite crystallization, which may attract the interest of research communities and expand the applications of IF-WS_2_@C nanoparticles in critical engineering.

## Author contributions

D. H. Cao proposed the ideas of ternary composites, designed the research and wrote the original draft. G. S. Liu, W. T. Chen and X. F. Lv made efforts on experimental testing sections. T. Z. Song, L. Y. Zhang, S. Liu and Y. Li provided necessary assistance. N. N. Wang and Y. Q. Zhu supervised the research and reviewed the draft.

## Conflicts of interest

The authors declare no conflicts of interest.

## Supplementary Material
